# Understanding the client characteristics of Aboriginal residential alcohol and other drug rehabilitation services in New South Wales, Australia

**DOI:** 10.1186/s13722-020-00193-8

**Published:** 2020-07-29

**Authors:** Douglas B. James, KS Kylie Lee, Tania Patrao, Ryan J. Courtney, Katherine M. Conigrave, Anthony Shakeshaft

**Affiliations:** 1 National Drug and Alcohol Research Centre, The University of New South Wales, Sydney, NSW 2052 Australia; 2 Faculty of Medicine and Health, Addiction Medicine, NHMRC Centre of Research Excellence in Indigenous Health and Alcohol, The University of Sydney, Camperdown, NSW Australia; 3 Centre for Alcohol Policy Research, La Trobe University, Bundoora, VIC Australia; 4 School of Public Health, University of Queensland, St Lucia, QLD Australia; 5 Royal Prince Alfred Hospital, Drug Health Services, Sydney Local Health District, Camperdown, NSW Australia

**Keywords:** Indigenous, Residential rehabilitation, Alcohol, Amphetamines, Drug

## Abstract

**Background:**

Aboriginal alcohol and other drug residential rehabilitation (residential rehabilitation) services have been providing treatment in Australia of over 50 years. However, there are no studies in Australia or internationally that document characteristics of clients attending Indigenous residential rehabilitation services worldwide. This is the first multi-site paper to describe key client characteristics of six Indigenous (hereafter Aboriginal Australians as the term recommended by the Aboriginal Health and Medical Research Council of New South Wales) residential rehabilitation services in Australia.

**Methods:**

All recorded client admissions between 1 January 2011 to 31 December 2016 were considered from six operating services in the Australian state of New South Wales. Data collected were classified into categories based on demographics, treatment utilisation, substance use, mental health and quality of life characteristics. Means, median and percentages were calculated (where appropriate).

**Results:**

There were 2645 admissions across the six services in the study period, with an average of 440 admissions per year across all services. Participants were aged between 26 to 35 years, with fewest participants aged 46 +. Program length ranged from 12 to 52 weeks (mean of 12 weeks). The completion rates and length of stay for each service ranged from less than two to more than 12 weeks. The principal drug of choice was alcohol and amphetamines in half of the services. Not all services used them, but a range of tools were used to measure treatment, substance use and mental health or quality of life outcomes.

**Conclusion:**

This study is the first internationally to describe the key features of multiple Aboriginal residential rehabilitation services. The variation in tools used to collect client data made it difficult to compare client characteristics across services. Future research could explore predictors of treatment completion, identify opportunities for standardisation in client assessments and validate cultural approaches of care. These efforts would need to be guided by Aboriginal leadership in each service.

## Background

Aboriginal and Torres Strait Islander Australians (herein referred to as ‘Aboriginal Australians’ as the term recommended by the Aboriginal Health and Medical Research Council for New South Wales [NSW] [1]) experience alcohol and other drug-related harms at disproportionately higher rates than non-Aboriginal Australians [2, 3]. These harms include psychological distress, suicide, dislocation, community and family violence and a lack of social capital [4, 5]. Young Aboriginal Australians (aged 15–29 years), compared to their non-Aboriginal counterparts, are up to five times more likely to experience hospitalisations or to die from an alcohol- or tobacco-related condition [6, 7]. Aboriginal Australians are overrepresented across all drug use categories as a percentage of the population in both drug treatment services. They use or consume drugs at rates 1.8 times higher than their non-indigenous counterparts. Aboriginal Australians also experience the impact of drug-related health problems at 2.3 times than their non-indigenous Australian counterparts [8].

Aboriginal community controlled alcohol and other drug residential rehabilitation (residential rehabilitation) services have provided treatment for Aboriginal people with substance use disorders for 50 years in NSW [9]. Within Australia, NSW is the state with largest population of Aboriginal Australians [10]. Aboriginal community controlled health services deliver holistic, comprehensive and culturally appropriate health care [11] that ensures treatment and service delivery are compatible with Aboriginal cultural beliefs and values [12].

Treatment programs and service delivery that are compatible with Aboriginal customs and beliefs, and that embed cultural values, have been shown to be of utmost importance to Aboriginal residential rehabilitation service providers, clients and other key stakeholders [13, 14]. However, there is also a need to ensure evidence-based practice in drug and alcohol treatment is provided. The Aboriginal Drug and Alcohol Residential Rehabilitation Network (ADARRN) is the peak organisation for Aboriginal residential rehabilitation services in NSW. This group has developed, in partnership with researchers, an evidence-based model of care by using three steps [13]. First, in-depth qualitative research was undertaken with clients, clinical staff and board members of one residential rehabilitation service to identify the core components of residential rehabilitation treatment perceived to be critical for an effective service [15]. Second, a systematic review of the existing research literature was undertaken to identify any components of Aboriginal residential rehabilitation services that have been shown to be associated with positive treatment outcomes [16]. Third, the results of the qualitative analysis and systematic review were combined to generate a best-evidence model of care for Aboriginal residential rehabilitation services in NSW [14]. The model of care was constructed as six core components that could be standardised across any Aboriginal residential rehabilitation (with cultural healing as the primary component) and activities to operationalise each core component [13, 16]. These activities can be tailored to the individual service and reflect the varying circumstances within each service. The model of care constructed was attuned to the need to balance standardised, best-evidence practice (the core components) with tailoring the delivery of those core components to the local circumstances of each service.

Having established a best-evidence and culturally safe model of care for Aboriginal residential rehabilitation services, it was then important for ADARRN to develop an evidence-based client assessment tool that could be integrated into routine clinical practice. This was important, firstly, because it provides the opportunity to understand the specific needs and risk factors of clients. This then creates the opportunity to tailor their treatment accordingly. One descriptive study of clients attending an Aboriginal residential rehabilitation service in NSW, for example, identified that three-quarters (77%) of clients were referred from the criminal justice system [17]. A possible consequence of this was that those clients may have less need for the management of any post-withdrawal syndrome complications, compared to self-referred clients whose recent use was likely to have been more frequent and heavier. Secondly, it provides the opportunity to directly measure outcomes and benefits of residential rehabilitation treatment for clients. A pre/post outcome study with one Aboriginal residential rehabilitation in NSW, for example, identified improvements in clients’ communication, conflict resolution and life skills [18]. This is opposed to studies that have used less direct, or proxy measures of treatment outcomes, such as longer time spent in treatment [19], or successful completion of a residential rehabilitation program [20]. The few studies that do report on services that used evidence-based outcome measures are limited to single sites, meaning their generalisability to other Aboriginal residential rehabilitation services is limited [16].

Consequently, this study is the first multi-site description of clients of Aboriginal community controlled residential rehabilitation services in Australia and internationally. It has two specific aims: (1) to describe the key characteristics of six Aboriginal residential rehabilitation services in NSW; and (2) to describe clients’ demographic, treatment utilisation, substance use, mental health and quality of life characteristics.

## Methods

### Ethics

Ethical approval was granted by the Human Research and Ethics Committee of the Aboriginal Health and Medical Research Council (No. 1227/16). Services involved in this research have given consent to be identified. This study was a retrospective study of de-identified service data (on individual clients) collected by 6 Aboriginal residential rehabilitation services in the Australian state of New South Wales (NSW). Furthermore, the study design was guided and endorsed by Aboriginal Residential Rehabilitation Healing Drug and Alcohol Network (ARRHDAN) the peak body governing Aboriginal residential rehabilitation services in NSW.

### Aboriginal leadership

Study methods were developed in consultation with ADARRN members. The study was led by an Aboriginal man (DJ) who has 30 years of clinical experience working in Aboriginal residential rehabilitation services in NSW.

### Settings

**Fig. 1:**
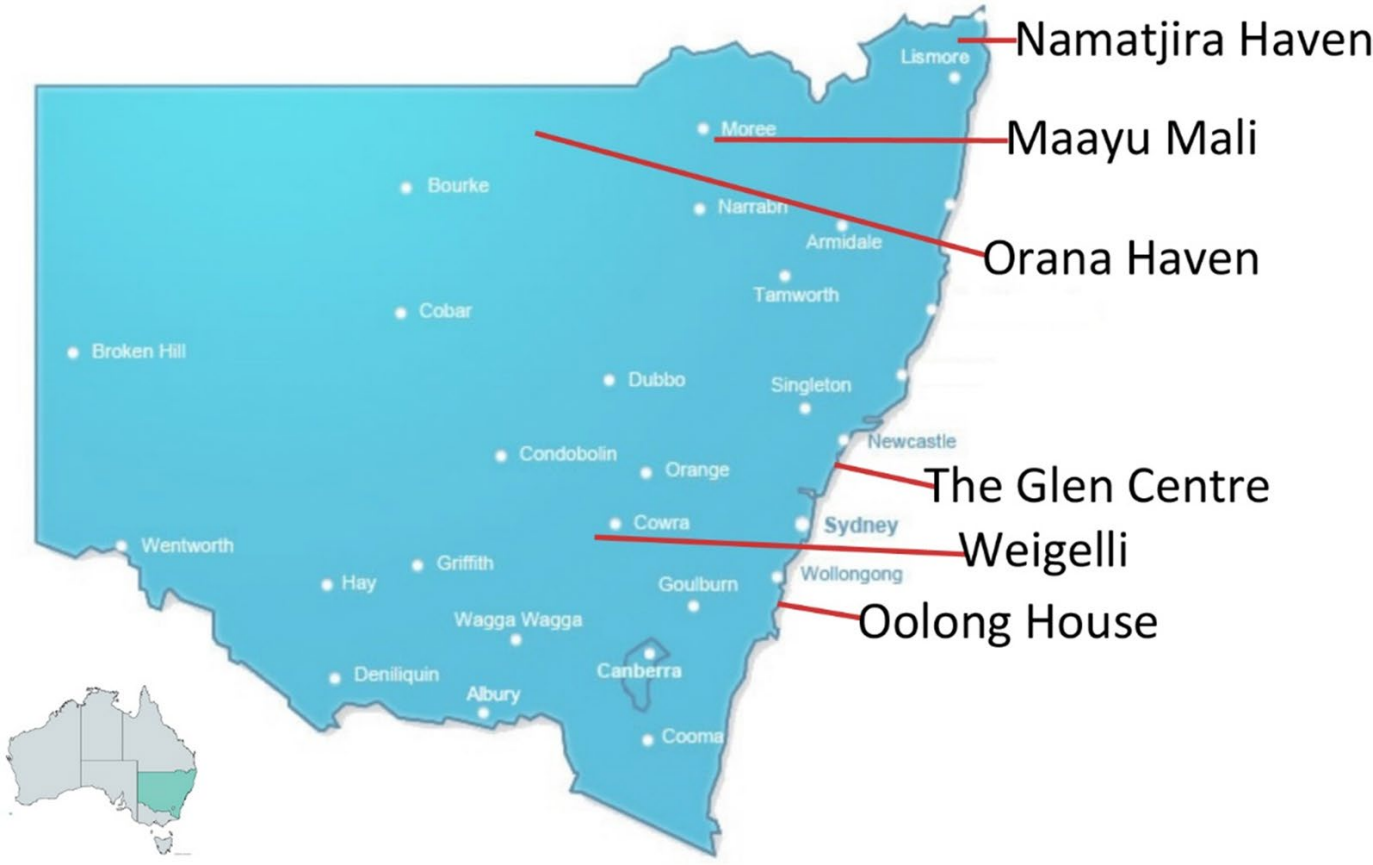
Map of six Aboriginal alcohol and other drug residential rehabilitation services in New South Wales Australia

The six currently operating Aboriginal alcohol and other drug residential rehabilitation services in NSW participated in this study. Each service is governed by an Aboriginal community controlled board of management. As shown in Fig. 1, all services are in regional or remote locations, between 180 and 650 km from Sydney (NSW), Australia. The names of each service (and of the traditional country on which it is located, and the number of years it has been operating) are: Namatjira Haven (Bundjalung, 26 years); The Glen Centre (Darkinjung, 26 years); Weigelli Centre (Wiradjuri, 21 years); Orana Haven Drug and Alcohol Rehabilitation Centre (Ngemba, 32 years); Oolong House (Dharrawal, 40 years) and Maayu Mali (Kamilaroi, 2 years).

### Sample

All recorded client admissions (intake data) were included from the six services from 1 January 2011 to 31 December 2016. For one service (Maayu Mali), data were only available from October 2015 (when this service commenced operations) to December 2016. Individual clients may have more than one episode of care which may occur over an extended time period. Each admission was therefore counted as unique for the purposes of analysis. All available data was used. Missing data was not imputed.

### Treatment program

#### Program pathways

**Fig. 2:**
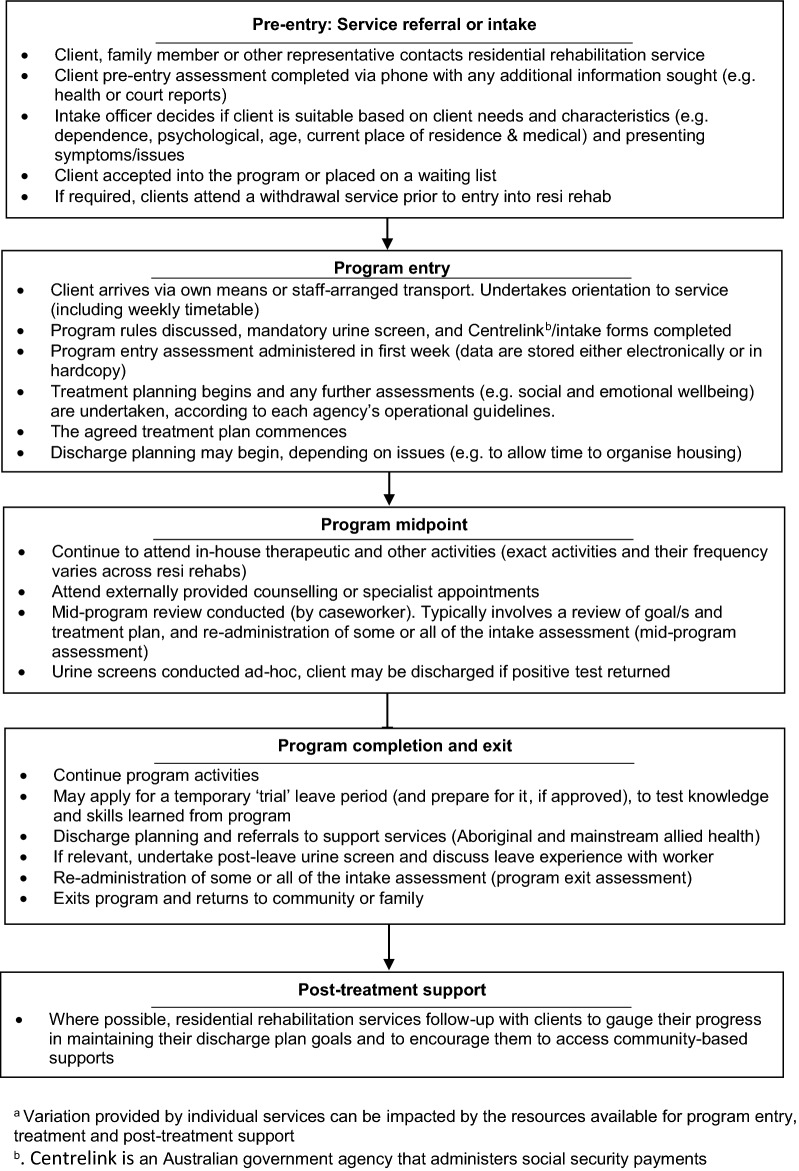
Typical pathway of care for clients of the Aboriginal alcohol and other drug residential rehabilitation services in New South Wales (NSW) Australia. Variation provided by individual services can be impacted by the resources available for program entry, treatment and post-treatment support. Centrelink is an Australian government agency that administers social security payments

A typical pathway of care is summarised in Fig. 2. For each residential rehabilitation service, clients are referred by an external organisation or agent, or by self-referral. A pre-entry assessment usually occurs before intake (typically by phone), and eligible applicants are invited to commence a residential program if a place is available, or otherwise clients are placed on a waiting list. Prior to program entry, it is a requirement of all services that clients attend detoxification (‘detox’; management of withdrawal syndrome). This could take the form a formal residential detox program or supervised in a hospital or community setting. Clients are required to be abstinent for program duration).These requirements to attend detox and be abstinent in the program were decided by the respective boards that govern each service at inception. At program entry a more detailed assessment is undertaken, and treatment planning starts. This includes assessment by an offsite or visiting medical officer. Treatment typically comprises implementation of an agreed care plan, a mid-program review, program completion and exit (including the development of a discharge plan) and, where possible, post-treatment support. Variation in treatment program activities, including the level of post-treatment support, reflects different levels of available resources for different services. Random urine screens are conducted in all services and a positive test may result in a client being discharged for a house rule violation. Urine screening is carried out on intake and ad-hoc during a program. Urine screenings are used only for the purposes of testing drug levels and or to inform reports for client court reports. Urine data was not made available to the research team.

### Measures

Data routinely collected by some or all services were classified into 12 categories: (1) demographics (age, sex, Aboriginal and Torres Strait Islander status, usual residential postcode, usual community of residence); (2) date of admission; (3) referral source; (4) length of stay; (5) discharge type (completed, self-discharge or house rules violation); (6) reason for attendance (principal/secondary substance of concern); (7) smoking status; (8) severity of drug or alcohol dependence (the 5-item Severity of Dependence Scale (SDS) [21] and the first seven items of the 13-item Indigenous Risk Impact Screen (IRIS) [22], which is a joint screening tool for alcohol and or drug disorders; (9) drug use disorders (the 11-item Drug Use Disorders Identification Test (DUDIT) [23]; (10) alcohol use and related harms (the 10-item Alcohol Use Disorders Identification Test (AUDIT) [24]; (11) mental health (the last 6 items of IRIS) [22] and the 10-item Kessler 10 (K10) [25] to assess psychological distress in the last 4-weeks); (12) Quality of Life (a World Health Organization tool (WHO-QoL) [26], that consists of a 26-item full questionnaire or an 8-item short-form). This tool measures quality of life in six domains—general quality of life, health, physical quality of life, psychological quality of life, satisfaction with social relationships, and satisfaction with the environment.

### Procedure

Each service used their own standard pen-and-paper intake (assessment) form on admission. Client details were recorded on an intake form and then entered into an electronic patient management system or stored in hardcopy. Data for one service were obtained from a paper by Munro and colleagues [17] because that service only had pen-and-paper records. Data for all services were de-identified prior to being exported into Microsoft Excel. Multiple Excel files for each service were then imported into Stata (Version 15) for analysis [27].

### Statistical methods

Extracted data were examined for outliers or inconsistencies. Data were then presented as descriptive statistics for demographic and treatment characteristics (Table 2), substance use characteristics (Table 3), and mental health and quality of life characteristics (Table 4).

#### Demographic and treatment characteristics

These comprise socio-demographics (age, Aboriginal status), length of stay, referral source and discharge type. As the first complete month of data were available from January 2011, calendar years were used to maximise the data included in analysis (1 January to December 31 for each year). Age was categorised as 18–25, 26–35, 36–45 and ≥ 46 years. Aboriginal status was dichotomised as ‘yes’ or ‘no’. Length of stay was categorised as less than 2 weeks; two to less than 4 weeks; four to less than 6 weeks; six but less than 8 weeks; and eight or more weeks. Referral source was categorised as criminal justice (on parole from incarceration or on bail), self or family or other (government or community-based agencies), and discharge type was categorised as: completed the program; self-discharge (against staff advice) or house rules violation.

#### Substance use characteristics

These consisted of principal and secondary substance of concern (categorised as alcohol, cannabis, amphetamines, opioids or other), smoking status in the last 12 months (categorised as current smoker, non-smoker or not stated), alcohol use risk (categorised using AUDIT cut-off scores: low-risk (0–7) [28], moderate-risk (8–15), high-risk (16–19), or further diagnostic evaluation required to investigate the possibility of alcohol dependence (20 or more) [28]; drug use risk (categorised using DUDIT cut-off scores: low-risk (0–24) or high-risk 25 +) [23]; and severity of alcohol and drug dependence (categorised using IRIS cut-off scores for being at risk score of 10 or more) [22] and SDS cut-off scores: low-risk (0–3), mild-risk (4–6), moderate-risk (7–9), substantial-risk (10–12) and severe-risk (13–15) [29].

#### Mental health and quality of life characteristics

Mental health risk is presented as psychological distress (categorised using K-10 cut-off scores: low (10–15), moderate (16–21), high (22–29) and very high (30 +) [25]; and risk of mental health problems (categorised using IRIS mental health cut-off score of 11 or more) [22]. Quality of life was quantified using the 8-item version of the WHO-QoL (the items which comprise the short-form were extracted, if the full 26-item WHO-QoL was done). Three sets of scores are presented (Table 4): (i) the mean and median total score for all six QoL domains (calculated using formulae provided by WHO) [29]; (ii) the index score (sum of raw scores from each of the 8-items), where a maximum score of 40 represents optimal quality of life; and (iii) mean and median score for each of the six domains of quality of life using formulae provided by WHO [29].

#### Data analysis

Completeness of data collection were examined. Incomplete datasets were not included in the study. Means, medians and percentages (where appropriate) were calculated to examine variation across residential rehabilitation services in relation to demographic and other client characteristics. Given the aims of this paper are to describe clients of Aboriginal alcohol and other drug residential rehabilitation facilities and identify opportunities for improving their data collection, no inferential statistical tests were performed.

## Results

### Key characteristics of Aboriginal residential rehabilitation programs

Table 1 shows the methods (and the approximate time point for each assessment): pre-entry (by phone prior to commencement); program entry (during week 1); mid-program (during weeks 4–8); and at program exit (during weeks 12–16). It also summarises the eligibility criteria for entry into each service (males/females/couples, aged 18 and older), the treatment options (program length, bed numbers and whether management of any withdrawal syndrome is provided), and the six core components of treatment and other health care that are standardised across all services in NSW [14]. Some activities are provided by the services themselves (e.g. therapeutic activities) and some by external agencies (e.g. literacy and numeracy courses as an education or life skills focussed core component). Relapse prevention pharmacotherapies may be prescribed during a client’s time in a detoxification unit. Relapse prevention pharmacotherapies are not offered in any of the six participating services. For aftercare planning and support, one service (The Glen) has a ‘transition back to community’ program of 3 months and follow-up support of up to 12 months. Three other services have planned follow-up support of up to 12 months (Weigelli Centre, Oolong House and Maayu Mali) which, in the Weigelli Centre, is provided by a dedicated after-care team. Two services provide opportunistic aftercare, such as relapse prevention phone support and referrals for housing and medical needs (Namatjira Haven, Orana Haven).

**Table 1:** Overview of the key service characteristics of the Aboriginal alcohol and other drug residential rehabilitation services in New South Wales (NSW), Australia

Key services provided	Study sites					
	Namatjira Haven	The Glen Centre	Weigelli	Orana Haven	Oolong House	Maayu Mali
Client assessments						
Pre-entry^a^	✓	✓	✓	✓	✓	✓
Program entry (week 1)^b^	✓	✓	✓	✓	✓	✓
Mid-program (weeks 4–8)^c^	✓ (week 6)	✓ (week 4)	✓(week 6)	✓(week 6)	✓ (week 4)	✗
Program exit (week 12–16)^d^	✓	✓	✓	✓	✓	✓
Eligibility	Males 18 years +	Males 18 years +	Males/females/couples 18 years +	Males 18 years +	Males 18 years +	Males/females 18 years +
Treatment options						
Length (weeks)	12 to 36	12	12	12 to 52	16+ (individual plans)	12
Bed numbers	16	20 (program) 18 (transition)	18	16	21	14 (males) 4 (females)
Manage withdrawal syndrome	✓ (2 beds)	✗	✗	✗	✗	✗
Treatment components and other health care						
1. Cultural healing^e^	✓	✓	✓	✓	✓	✓
2. Case management	✓	✓	✓	✓	✓	✓
3. Education/life skills	✓	✓	✓	✓	✓	✓
Other outside programs	✓	✓	✓	✓	✓	✓
4. Therapeutic activities:						
Group work	✓	✓	✓	✓	✓	✓
Counselling (SEWB support)^f^	✓	✓	✓	✓	✓	✓
Physical health check (GPs)	✓	✓	✓	✓	✓	✓
5. Time out from substances	✓	✓	✓	✓	✓	✓
6. Aftercare planning/support^g^	(Opportunistic)^h^	(Transition, 3–12 months)^i^	(Planned to 6-months)^j^	(Opportunistic)	(Planned to 12-months)	(Planned to 12-months)

^a^Initial assessment: occurs when clients first make contact with service; typically done by phone

^b^Week 1: Client enters service, undertakes orientation to service and health checks (medical), care plan is developed

^c^Week 4–8: Progress reviews typically occur at Week 6 but can be conducted when needed

^d^Week 12: Program exit assessment (at program completion) typically at Week 12, except for Oolong House where exit interviews occur in Week 16

^e^All services have cultural healing and safety embedded in their program, including regular cultural activities (e.g. learning culture and language)

^f^Social and emotional wellbeing programs are typically run by Aboriginal mental health workers, with counsellors and other health professionals

^g^Aftercare planning typically begins at Week 8 but can occur earlier if needed (e.g. accommodation post-treatment)

^h^Opportunistic refers to when a service is able to allocate time to undertake after care support

^i^Transition refers to when a client is moves between a residential client into the community

^j^Planned refers to a planned systematic approach to aftercare that begins whilst a residential client

### Demographic and treatment characteristics

There were 2645 admissions across the six residential rehabilitation services over 6 years, with an average of 440 admissions per year across all services (Table 2). The mean age of clients ranged from 32 to 35 years. Most participants were aged between 26 to 35 years, with fewest participants aged older than 46. Program length ranged from 12 to 52 weeks (mean of 12 weeks). Referrals from the criminal justice system were comparable across three services (24–28%) and lower in a fourth service (16%). Self-referrals were similar for two services at around 40%, lower in one service (29%) and higher in a fourth service (60%). Referrals from other sources (such as government and non-government service providers) were comparable for two services (34% and 33%) and substantially different for another two services (12% and 56%).

**Table 2:** Demographic and treatment characteristics of clients attending an Aboriginal alcohol and other drug residential rehabilitation service in New South Wales (NSW), Australia (1 January 2011–31 December 2016)

Demographic and treatment characteristics Admissions (n)	Study sites (N = 2645)					
	Namatjira Haven	The Glen Centre	Weigelli	Orana Haven	Oolong House	Maayu Mali
	n = 382	n = 798	n = 589	n = 329	n = 344	n = 203
Socio demographics						
Age (mean)^a,b^	35	33	32	34	34	33
Age groups (in years; %)						
18–25	20	25	28	25	25	22
26–35	34	35	38	36	34	41
36–45	27	29	26	26	27	24
≥ 46	19	12	8	13	14	13
Aboriginal or Torres Strait Islander (%)	92	59	83	85	62	–^c^
Length of stay (%)^d,e^						
Less than 2 weeks	14	18	21	20	33	–
2 to less than 4 weeks	12	11	20	15	9	–
4 to less than 6 weeks	24	18	14	11	13	–
6 to less than 8 weeks	30	32	8	5	7	–
8 + weeks	20	21	37	49	38	–
Referral source (%)						
Criminal justice system	16	28	24	–	28	–
Self	29	38	43	–	60	–
Other	56	34	33	–	12	–
Discharge type (%)						
Completed^f^	–	55	–	33	30	–
Self-discharge	–	44	–	47	40	–
House rules violation	–	1	–	20	30	–

^a^Data available for n = 788 (The Glen Centre)

^b^Data available for n = 327 clients (Orana Haven)

^c^Represents data not available

^d^Data available for n = 233 clients (Namatjira Haven)

^e^Data used from a paper by Munro and colleagues (2018; Orana Haven)

^f^Program completion is 12 weeks (for five services) and 16 weeks (for Oolong House)

The proportion of clients who completed a program varied from 30% (Oolong House) to 55% (The Glen Centre). Self-discharge rates were comparable across all services, ranging from 40 to 47%. Discharge because of house rules violations were comparable for two of the three services that presented these data (20% and 30%).

### Substance use characteristics

#### Principal and secondary substance of concern

As summarised in Table 3, the most common primary substance of concern was alcohol in three services (Namatjira Haven, 66%; Maayu Mali, 41%; Oolong House, 48%) and amphetamines in the other three services (The Glen Centre, 45%, Weigelli Centre, 41%, Orana Haven 63%). For the two services that assessed secondary substance of concern, the most common substance was cannabis in one service (Namatjira Haven, 53%) and alcohol in the other (Weigelli Centre, 34%).

**Table 3:** Substance use characteristics of clients attending an Aboriginal alcohol and other drug residential rehabilitation service in New South Wales (NSW), Australia (1 January 2011–31 December 2016)

Substance use characteristics	Study sites (N = 2104)					
	*Namatjira Haven*^a^ *n* = *349*	*The Glen Centre*^b^ *n* = *703*	*Weigelli* *n* = *589*	*Orana Haven*^c^ *n* = *51*	*Oolong House* *n* = *344*	*Maayu Mali* *n* = *68*
Principal substance of concern (%)						%
Alcohol	66	36	29	16	48	41
Cannabis	3	7	14	20	12	16
Amphetamines	18	45	41	63	32	39
Opioids	13	8	9	1	5	3
Other	–	4	7	–	3	1
Secondary substance of concern (%)						
Alcohol	7	–	34	–	–	–
Amphetamines	34	–	27	–	–	–
Opioids	5	–	11	–	–	–
Cannabis	53	–	26	–	–	–
Other	1	–	2	–	–	–
Smoking status (%)						
Smoker (in the last 12 months)	56	82	84	81	–	–
Non-smoker (in the last 12 months)	37	18	6	19	–	–
Not stated	7	–	10	–	–	–
Alcohol use risk: 10-item AUDIT (%)						
Low risk (doug0–7)	20	–	–	–	–	–
Moderate risk (8–15)	10	–	–	–	–	–
High risk (16–19)	11	–	–	–	–	–
Dependent (20 +)	59	–	–	–	–	–
Drug use risk: 10-item DUDIT (%)						
Low risk (score 0–24)	12	–	–	–	–	–
High risk (score > 25)	88	–	–	–	–	–
Severity of dependence (%) (Indigenous Risk Impact Screen—IRIS)						
At risk (score 10+)	98	–	–	–	–	–
(Severity of Dependence Scale—SDS)						
Low (score 0–3)	–	4	10	–	–	–
Mild (score 4–6)	–	13	25	–	–	–
Moderate (score 7–9)	–	28	28	–	–	–
Substantial (score 10–12)	–	35	21	–	–	–
Severe (score 13–15)	–	20	16	–	–	–

^a^Data varied for numbers of clients for different measures (range: n = 41 to n = 149 for Namatjira Haven)

^b^Data varied for numbers of clients for different measures (range: n = 703 for The Glen Centre, except for smoking status n = 133)

^c^Data used from a paper by Alice Munro and colleagues (2018; Orana Haven)

#### Tobacco

For the four services that collect these data, smoking rates were over 80% for three services, with more than half of clients (56%) smoking in the fourth (Namatjira Haven).

#### Alcohol and other drug use and dependence

In the only service that used AUDIT [28] and DUDIT [23] (Namatjira Haven), seven out of 10 clients were categorised on AUDIT as being high-risk (11%) or likely dependent (59%), and 88% of clients were categorised as being at high-risk for dependence on drugs. For the only service that used the IRIS [22] (Namatjira Haven) nearly all clients (98%) were categorised as being at high-risk of substance use dependence (score ≥ 10). For the two services that used SDS [29], the majority of clients were categorised at being at moderate to severe risk of substance use dependence (The Glen Centre, 83%; and Weigelli Centre, 65%).

### Mental health and quality of life characteristics

As summarised in Table 4, three services used K10 (Namatjira Haven, The Glen Centre and Weigelli Centre). An average of eight out of ten (78%) clients experienced moderate to very high psychological distress. Almost twice as many clients were at high or very high-risk of psychological distress at Namatjira Haven (70%) and Weigelli Centre (71%), compared to The Glen Centre (38%). Only Namatjira Haven used IRIS [22] and nearly nine out of ten (88%) clients were categorised as being at-risk of a mental health problem.

**Table 4:** Mental health and quality of life characteristics of clients attending an Aboriginal alcohol and other drug residential rehabilitation service in New South Wales (NSW), Australia (1 January 2011–31 December 2016)

Mental health and quality of life characteristics	Study sites (N=2422)					
	Namatjira Haven^a^	The Glen Centre^b^	Weigelli	Orana Haven	Oolong House	Maayu Mali
	*n* = *200*	*n* = *703*	*n* = *589*			
Mental health (%)						
Kessler-10 (%)	–	–	–	–	–	–
Low psychological distress (score 10–15)	13	39	12	–	–	–
Moderate psychological distress (score 16–21)	17	23	17	–	–	–
High psychological distress (score 22–29)	28	19	35	–	–	–
Very high psychological distress (score > 30)	42	19	36	–	–	–
Indigenous Risk Impact Screen (IRIS)—mental health and wellbeing						
At risk (score 11 +)	88	–	–	–	–	–
Quality of life (%)						
WHO-Quality of Life (8 item version)^c^						
Total score for all 6 domains: mean, median	26, 26	30, 31	–	–	–	–
Index score (raw score for each of the 8 items):	25	30	–	–	–	–
Individual domain scores: mean, median						
1. Quality of life	5.9, 6.0	8.3, 8.0	–	–	–	–
2. Health	5.9, 6.0	7.3, 8.0	–	–	–	–
3. Physical quality of life	6.7, 7.0	8.1, 8.0	–	–	–	–
4. Psychological quality of life	6.5, 6.0	7.5, 8.0	–	–	–	–
5. Satisfaction with social relationships	6.3, 6.0	7.0, 8.0	–	–	–	–
6. Satisfaction with environment	6.3, 7.0	7.4, 8.0	–	–	–	–

^a^Data varied for numbers of clients for different measures (range: n = 119 to n = 200; for Namatjira Haven)

^b^Data varied for numbers of clients for different measures (range: n = 703 for The Glen Centre)

^c^Namatjira Haven uses the 26-item WHO-Qol while The Glen Centre uses the 8-item, which is a subset of the longer 26-item instrument. To assist with comparability, only the subset of 8-items collected by both services were considered

Clients’ mean and median total score for all six quality of life [29] domains were slightly higher in The Glen Centre compared with Namatjira Haven (the only two services that collected this data; Namatjira Haven: mean = 26, median = 26; and The Glen Centre: mean = 30, median = 31). Total quality of life index scores (across all six domains) were also higher for clients of The Glen Centre compared to Namatjira Haven (30 versus 25).

## Discussion

To the authors’ knowledge, based on our 2017 systematic review of indigenous drug and alcohol residential rehabilitation services [16], this study is the first internationally to describe the key features of multiple Aboriginal residential rehabilitation services. The results show both consistency and substantial variation across services.

### Similarities across Aboriginal residential rehabilitation services

The typical location, pathways of care from pre-entry through to program completion including post-treatment support, and the key service characteristics of Aboriginal residential rehabilitation services in NSW are similar. They are all based in regional, rural or remote locations rather than in the larger metropolitan centres. Given the majority of Aboriginal people in NSW live in cities, there may be benefit in establishing an urban Aboriginal residential rehabilitation that can focus on cultural healing [17]. Similarly, given only two services allow females and only one service allows couples, there appears to be scope for at least one female-only residential rehabilitation service, and a couples or family-focused residential rehabilitation. Evidence from non-Indigenous residential rehabilitation services suggests that female-specific [30] and family-focused [31] services are in demand [32] and can be effective.

The mean age of clients was comparable (ranging from 32 to 35 years) with the highest proportion of clients aged 26–35 years (range: 34–41%). Alcohol and amphetamines were easily the most common primary substances of concern (range: 41–66%). These characteristics are similar to other treatment services across Australia [33]. Given these data are for the period 2011 to 2016, and with recent trends for increased amphetamine and opioid use among illicit drug users in Australia [34], the proportion of clients with amphetamine or opioids as their primary drug of concern may have increased since 2017. The variation in principal substances of concern are likely due to availability and local supply networks particularly in rural and remote locations.

A majority of clients were smokers (range: 56–84%), which is higher than rates for other Aboriginal Australians and significantly higher than the broader Australian population (12%; aged 14 +) [35]. There is evidence that clients of alcohol and other drug services would consider quitting tobacco [36, 37]. The development and incorporation of smoking cessation treatment and its integration in the Aboriginal residential rehabilitation setting requires further consideration and action [38]. There is firm evidence that pharmacotherapies are useful in treating alcohol and other drug use disorders [39–41]. However, the six services involved in this study do not have capacity to provide pharmacotherapies. This is due to lack of access to trained staff to administer these medications. Current efforts are underway to employ qualified health and medical staff to provide pharmacotherapies in Aboriginal residential rehabilitation services.

### Variation across Aboriginal residential rehabilitation services

In terms of variation across services, the proportion of clients who identified as Aboriginal ranged from 59 to 92%. This variation is most likely a consequence of individual service’s operating guidelines. These guidelines determine the extent to which each Aboriginal-led service may engage with non-Aboriginal clients and family members. One-third of services (n = 2/6) do not accept criminal justice referrals. This was decided at each service’s inception; however, it may mean that a particularly high-risk group is being missed by these services. Previous studies have found that clients referred from the criminal justice system (16% to 28% of clients in the current study) are most vulnerable in the period immediately following release from prison, with high risk of overdose and dropout [42, 43],. This highlights the need for targeted services for this population [44]. It may also provide an opportunity for the two Aboriginal residential rehabilitation services that do not currently accept referrals from the criminal justice program to revisit their intake procedures. Given the unique characteristics of clients exiting the criminal justice system, compared to self-referrals, services might also consider adding activities to their core model of care to better meet their needs. For example, adding programs on anger management, cultural identity, parenting, relationships and self-awareness.

It is not clear why variation in psychological distress exists (19% for The Glen Centre versus 36% for Weigelli Centre and 42% for Namatjira Haven). There could be several reasons for this variation. Firstly, fewer clients were admitted to the Glen Centre who are Indigenous (59%) compared with Namatjira Haven and Weigelli Centre (92% and 83%). National surveys have reported higher levels of psychological distress among Aboriginal people compared with their non-Indigenous Australian counterparts [45] (26.6% versus 13.1%). Also, the Glen Centre had nearly double the proportion of client referrals from criminal justice compared with Namatjira Haven that had the lowest (28% versus 16%). It is possible that clients entering The Glen Centre have had sufficient time already (in prison) to work through residual withdrawal from alcohol and/or other drugs. In contrast, clients admitted to Namatjira Haven could be experiencing withdrawal at the time of intake (and hence likely to have poorer psychological distress scores).

K10 [29] is widely used in Australia and internationally for non-Indigenous populations. However, it has not been validated for an Indigenous Australian audience. The Indigenous Risk Impact Screening [22] tool has been validated for use with Aboriginal people in Australia and was used by one service (Namatjira Haven). Using IRIS (mental health), nearly nine out of ten clients experienced high levels of psychological distress.

Across the six services, length of stay varied from less than 2 weeks, to 8 weeks or more (Table 2) and up 1 year in some individual [rare] circumstances. Length of stay for Aboriginal clients can be affected by factors including principal drug of choice, interpersonal factors, a strong sense of culture and negative relationships with staff members [46, 47]. Further research could examine characteristics (on intake and exit) that predict length of stay and why people stay longer or leave early. To enable a more individualised approach, treatment length could also be tailored to each client, be agreed on at intake assessment, and monitored during treatment [48, 49]. Each service offers a range of similar programs that are tailored to local operating guidelines. Research is being undertaken across the six services to consider the role for individual client-focused programs and processes [50]. From there, a more individual approach could be offered to cater for specific cultural, social and emotional needs of each client.

A range of measures were used to document client progress in each service. Mental health data, for example, was collected by three services though the measures used varied (IRIS for Namatjira Haven; K10 for Namatjira Haven, The Glen Centre and Weigelli Centre; and WHO-Quality of Life for Namatjira Haven and The Glen Centre). Standardising data collection would enable comparison across services and help staff monitor treatment and after-care outcomes [20, 51, 52]. Although all services have a focus on data quality, a lack of opportunities to upskill staff in recording client information and staff time pressures led to substantial variation in record keeping. In keeping with this, adequate resourcing could be provided to employ dedicated continuous quality improvement personnel to monitor and support data quality. A schedule of staff training may assist to increase data completeness and inform continuous quality improvement [52]. The use of systematically collected and standardised data would inform practice (e.g. help to individualise treatment provided) and could also be used to justify required funding increases [53, 54].

In addition to standardising data collection across services, this study found no measure of the role of culture in treatment, despite cultural connection and safety being identified as central to each service’s model of care [13, 15], Internationally, studies of Indigenous residential rehabilitation services in the US, for example, identified cultural practices (e.g. sweat lodges) in recovery that are important to indigenous peoples [46]. Being able to measure the value of culture in Aboriginal rehabilitation services could help corroborate the connections between culture, treatment and recovery [51, 55].

### Study limitations

Data collection across each service varied from paper-and-pen intake forms to the use of patient software systems. This may have impacted on the quality and comprehensiveness of the data that were collected between services and across each client’s treatment program. Just one measure (IRIS) has been validated for use with Indigenous Australians (in Queensland) [22], which may suggest that some data in this analysis are not accurate or reliable for Indigenous clients The last data point in this study was 2016, so there may have been changes in outcomes and data collection efforts since that time.

## Conclusion

This study represented six Aboriginal residential rehabilitation services delivering treatment programs using the same core and other health care components but delivering those core components according to their own unique guiding philosophies. However, variation in tools used to collect client data made it difficult to compare a full range of client characteristics across services. More research is needed to explore predictors of treatment completion, to identify opportunities for greater standardisation in client assessments and to validate cultural approaches of care offered in this sector. This research could look at the relationship between client and service characteristics and treatment outcomes including length of stay. This information could help inform program and policy development that is resource efficient as well as client centred. There might also be benefit in the services developing a more structured approach to include manualised behavioural interventions in their program planning and development. These efforts would need to be guided by local Aboriginal leadership of each service.

## Data Availability

Data for this project is stored at the University of NSW based at National Drug and Alcohol Research Centre, 22-32 King Street Randwick New South Wales, 2031 Australia

## Abbreviations

ADARRN: Aboriginal Drug and Alcohol Residential Rehabilitation Network
NHMRC: National Health and Medical Research Council
DJ: Doug James
NSW: New South Wales
